# Optical Biosensors Based on Silicon-On-Insulator Ring Resonators: A Review

**DOI:** 10.3390/molecules24030519

**Published:** 2019-01-31

**Authors:** Patrick Steglich, Marcel Hülsemann, Birgit Dietzel, Andreas Mai

**Affiliations:** 1 IHP—Leibniz-Institut für Innovative Mikroelektronik, Im Technologiepark 25, 15236 Frankfurt (Oder), Germany; mai@ihp-microelectronics.com; 2 Technische Hochschule Wildau, Hochschulring 1, 15745 Wildau, Germany; dietzel@th-wildau.de; 3 First Sensor AG, Peter-Behrens-Straße 15, 12459 Berlin, Germany; marcel.huelsemann@first-sensor.com

**Keywords:** biosensors, biophotonics, integrated optical sensors, aptamers, biomaterials, optical sensor, silicon photonics, ring resonators, lab-on-a-chip

## Abstract

Recent developments in optical biosensors based on integrated photonic devices are reviewed with a special emphasis on silicon-on-insulator ring resonators. The review is mainly devoted to the following aspects: (1) Principles of sensing mechanism, (2) sensor design, (3) biofunctionalization procedures for specific molecule detection and (4) system integration and measurement set-ups. The inherent challenges of implementing photonics-based biosensors to meet specific requirements of applications in medicine, food analysis, and environmental monitoring are discussed.

## 1. Introduction

Silicon-based photonic biosensors integrated into a semiconductor chip technology can lead to major advances in point-of-care applications, food diagnostics, and environmental monitoring through the rapid and precise analysis of various substances. In recent years, there has been an increasing interest in sensors based on photonic integrated circuits (PIC) because they give rise to cost effective, scalable and reliable on-chip biosensors for a broad market [1,2].

The silicon-on-insulator (SOI)-technology is the most attractive technology for PICs since it provides a scalable platform for mass production [3,4] and the opportunity for monolithic integration of electronic and photonic devices, which is known as electronic photonic integrated circuits (EPIC) [5]. This allows the integration of sensors, detectors, light-sources and read-out electronics in a single chip [6,7,8,9,10].

Once the photonic chip is fabricated, the silicon surface of the sensor can be coated with a covalently attached sensing layer [11]. This layer determines the specific detection and, hence, the application. This step, however, is independent from the fabrication of the chip, making the SOI-technology attractive for both, science and industry. A further advantage of SOI-based biosensors is the possibility to realize sensor arrays. This allows for the detection of several substances in parallel (multiplexing) [12].

The SOI-based photonic biosensor can be realized by using interferometric or resonant structures. The former one is usually based on a Mach-Zehnder interferometer [13] configuration and the later most often on a ring resonator [14]. Mach-Zehnder interferometer have a higher temperature stability compared to ring resonators but suffer from high sensitivity [15,16,17]. Ring resonators, however, have the advantage over Mach-Zehnder interferometer of a high sensitivity and small footprint, which allows for a dense sensor-integration [18]. It is notable that, beside aforemementioned SOI-based sensors, plasmonic sensors have gained increasing research interest [19,20,21]. Those sensors were demonstrated to achieve extraordinary high sensitivity but have the disadvantage to be not yet compatible with complementary metal-oxide semiconductor (CMOS)-based microelectronics for a direct readout.

Competitive traditional biosensing techniques that are already commercially used are, for example, enzyme-linked immunosorbent assay (ELISA) [22], electrochemical sensor [23], bilayer lipid membranes (BLM) [24], high performance liquid chromatography (HPLC) [25,26], micro-electrode immunoassay [27], field immunoassay [28] and surface plasmon resonance spectroscopy (SPR) [29,30]. Compared to those traditional benchtop electrochemical instruments, the main advantages of SOI-based ring resonators are their small size, scalable mass-production and fast readout (real-time) as well as the possibility for low cost, portable devices for point-of-care applications since they rely on CMOS-compatible processes.

In this work, we focus on SOI-based ring resonators and provide in the first part an overview of the working principle and sensing mechanism. The detection limits and integration challenges are critically discussed. In the second part, we review recent advances on surface functionalization and report on the detection of various biomarkers. Finally, we discuss typical experimental set-ups and recent developments regarding integration approaches.

## 2. Photonic Devices and Sensing Mechanisms

This section provides an introduction to photonic biosensors based on SOI ring resonators and to the underlying sensing mechanism. Furthermore, we discuss recent advances in device design and operation principles.

### Operation Principle

**Figure 1:**
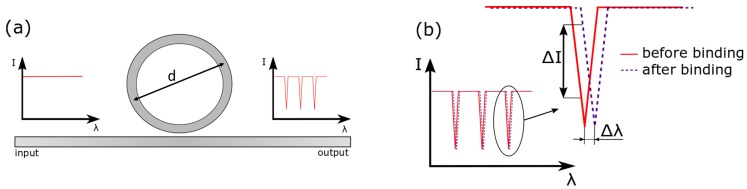
(**a**) Schematic representation of a silicon-on-insulator ring resonator. According to the resonance condition, only selected wavelengths can propagate in the ring and distinct resonance peaks appear in the output spectrum. Typical ring diameter *d* range from 20 μm to 100 μm. (**b**) Molecular binding takes place if a sample of the analyte gets in touch with the adsorbed layer on top of the silicon waveguide leading to a resonance wavelength shift Δλ.

This work is focusing on photonic sensors based on optical ring resonators in a SOI-technology. A ring resonator is composed of a silicon-based waveguide on top of a buried oxide substrate. In general, the light of a tunable laser or a superlumineszenzdiode is coupled to the waveguide via grating coupler or by butt coupling. The light is then partly coupled to the ring resonator if the resonance condition is fulfilled leading to resonance peaks at the output spectrum, as illustrated in Figure 1a. At the output, the light is coupled to a photodetector or an optical spectrum analyzer depending on the light source. Current advances in heterogeneous as well as monolithically integration give rise to implement laser [31,32] and photodiodes [33] on the same chip together with the photonic sensors.

After the fabrication of the chip, the surface of the silicon ring resonator is functionalized with an adsorbed layer for specific detection [34]. Here, the natural silicone oxide layer on top of the silicon waveguide is advantageously used. Molecular binding takes place if a sample of the analyte gets in touch with the adsorbed layer on top of the silicon waveguide. This results in a resonance wavelength shift, as shown in Figure 1b.

However, the general sensing mechanism underlying their operation is evanescent field sensing. If the evanescent field is altered due to an immobilization of analytes on the silicon waveguide, the resonance condition of the ring resonator is changed leading to a resonance wavelength shift. In this way, antibodies that only attach to their corresponding antigens are detected with high specificity by detecting either the resonance peak shift Δλ or the intensity change ΔI (see Figure 1b). Typical operation wavelengths are within the optical O-band (λ=1310nm) in the optical C-band (λ=1550nm) because silicon is highly transparent at these wavelengths. A second reason is that these wavelengths are widely used in telecommunication applications and, therefore, many low-cost laser sources are available [35,36]. Once the analyte-antibody binding took place, the residuals can be removed by drying or flushing to enhance the specific measurement. The wavelength shift can be calculated from resonator metrics that appear in [37]

(1) $$\Delta \lambda =\frac{\lambda_{res}}{n_{g}}\Delta n_{clad},$$

where $n_{g}$ is the group index, $\lambda_{res}$ is the resonance wavelength and $\Delta n_{clad}$ determines the refractive index change of the cladding material. The group index can be calculated by

(2) $$n_{g}=n_{eff}-\lambda \frac{\delta n_{eff}}{\delta \lambda }.$$

Here, the effective refractive index of the optical waveguide is denoted as $n_{eff}$. The second term of Equation (2) is referred to as dispersion of the group index. Assuming a small resonance wavelength shift and, hence, a flat dispersion, the group index can be approximated by

(3) $$n_{g}\approx n_{eff}=\frac{\lambda^{2}}{FSR}\frac{1}{L_{ring}},$$

where $L_{ring}$ is the ring circumference and FSR is the free spectral range.

**Figure 2:**
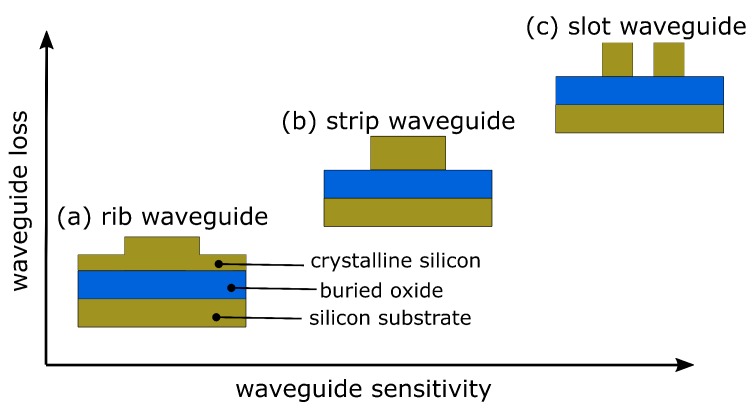
Typical silicon-on-insulator waveguide geometries for optical biosensing.

**Figure 3:**
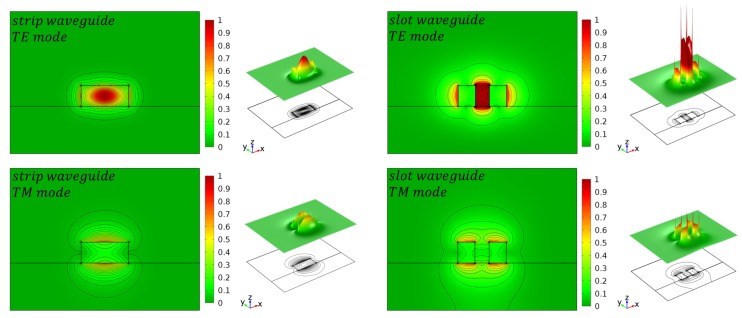
Simulation of the normalized E-field intensity for the first TE- and TM-mode for a strip and slot waveguide. Reproduced from Ref. [38] (CC BY 4.0).

The most important component of all integrated photonic biosensors is the silicon waveguide. During the last decade, many effort has been undertaken to improve waveguide geometries for optical sensing by simulation studies [38,39,40]. In principle, there are three types of widely used waveguides, namely strip waveguide, rib waveguide and slot waveguide, as illustrated in Figure 2. The evanescent field of the guided mode is partially penetrating into the cladding material, where the analyte is located. The amount of light penetrating into the cladding is different for each waveguide configuration and correlates with unwanted optical losses; i.e., the more light is penetrating into the cladding the higher the optical losses due to absorption and scattering. For example, the light is mainly confined inside the silicon core in case of a strip waveguides but in case of slot waveguides the light can be significantly confined in the vicinity of two silicon rails, as illustrated in Figure 3.

Depending on the application, it is necessary to choose an appropriate waveguide type. Rib waveguides show low optical losses at the cost of sensitivity. In contrast, slot waveguides exhibit a large sensitivity but high optical loss at the same time. Strip waveguides, in contrast, offer a good compromise between loss and sensitivity, as illustrated in Figure 2. As a rule of thumb, the more light is interacting with the analyte the higher is the waveguide sensitivity but the optical losses are increased, too. From Figure 3 it is apparent that the highest optical field confinement in the cladding is provided by the slot waveguide structure. Simulation studies have revealed that SOI slot waveguides achieve an optical field confinement of 0.7, i.e., 70% of the guided light is confined in the cladding and not in the silicon core [39,40,41]. In contrast, strip waveguides achieve an optical field confinement factor of 0.2. Another important parameter is the polarization state of the light, which is usually either transverse-electric (TE) or transfers-magnetic (TM). Most often, the guided light is TE-polarized in SOI-based PIC because it provides less optical losses. On the other hand, TM-polarized light can lead to an increased sensitivity since it has a higher field overlap with the cladding material, where the analyte is located. However, TE-polarized light is presumed in this work, except something else is specified. A comprehensive design guideline to choose the most appropriated waveguide type for a specific application can be found in Refs. [42,43]. To improve the sensor performance in terms of sensitivity, it is useful to distinguish between the waveguide sensitivity and the ring resonator sensitivity. The former describes the interaction of the guided light with the surrounding medium. It takes into account that the effective refractive index is altered if the cladding refractive index is changed. The waveguide sensitivity is given by

(4) $$S_{wg}=\frac{\Delta n_{eff}}{\Delta n_{clad}},$$

where $\Delta n_{eff}$ represents the effective refractive index change. Such a definition is useful for waveguide optimization through simulation studies. However, the ring resonator sensitivity depends not only on the waveguide geometry and, therefore, a second definition defining the ring resonator sensitivity is given by

(5) $$S_{rr}=\frac{\Delta \lambda }{\Delta n_{eff}}.$$

Taken both definitions into account, we get the overall photonic device sensitivity defined by

(6) $$S=S_{wg}S_{rr}=\frac{\Delta n_{eff}}{\Delta n_{clad}}\frac{\Delta \lambda }{\Delta n_{eff}}=\frac{\Delta \lambda }{\Delta n_{clad}}.$$

Please note that the unit of this definition is often written as nm/RIU, where RIU denotes the refractive index unit. It should be noted that the change of the cladding refractive index $\Delta n_{clad}$ is induced by binding of antigens to the functionalized waveguide surface. These definitions, however, are solely related to the photonic device and not to a directly measurable quantity. In this scenario, the minimum detectable change in the cladding refractive index gives us the limit of detection (LOD), which depends clearly on the minimum detectable resonance wavelength shift $\Delta \lambda_{min}$ that can be resolved by the measurement set-up. For example, an optical spectrum analyzer has a typical wavelength resolution of 20pm. This measurement resolution (MR) can be determined by

(7) $$MR=\Delta \lambda_{min}.$$

This leads to the LOD given by

(8) $$LOD=\frac{\Delta \lambda_{min}}{S}.$$

To get a metric which is independently from the measurement set-up, an intrinsic LOD (iLOD) is necessary [44]. It can be obtained by setting the measurement resolution MR as full width at half maximum (FWHM) of the resonance peak, which leads to

(9) $$iLOD=\frac{FWHM}{S}=\frac{\lambda_{res}}{QS},$$

where *Q* denotes the optical quality factor, which is determined by

(10) $$Q=\frac{\lambda_{res}}{FWHM}.$$

Finally, we provide a strategy to improve the waveguide geometry by design. Towards this, we consider the most important characteristics of integrated photonic biosensors, which can be divided into five categories [14]:

1. Increasing the waveguide sensitivity $S_{wg}$ increases the light-analyte-interaction. In fact, this determines the wavelength shift Δλ and has a strong impact on the overall sensitivity.
2. Enhancing the ring resonator sensitivity $S_{rr}$, which determines the wavelength shift Δλ depending on the refractive index change $\Delta n_{eff}$. This can be achieved by increasing the light-matter interaction using slot waveguide structures.
3. A small FWHM, i.e., a high Q-factor, impacts the sensitivity of ring resonator sensors since the impact of noise on the determination of the resonance wavelength will be reduced [45,46]. A higher Q-factor leads to a lower attenuation in the ring and minimizes the smallest detectable wavelength shift Δλ and consequently the detection limit.
4. A small footprint is directly related to the detection time and reduces the area consumption and therefore device costs significantly. Furthermore, this allows a high integration density, which is of special interest for multiplexing.
5. Compatibility with a semiconductor production platform, which gives the ability for an industrial production flow. The compatibility with an electronic-photonic integrated circuit (EPIC) allows for a monolithic integration.

**Figure 4:**
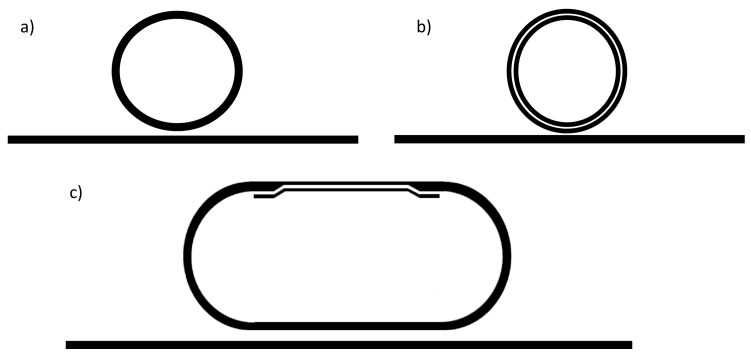
Schematics of different ring resonator concepts: (**a**) Common silicon strip-waveguide ring resonator. (**b**) Fully slotted ring resonator with a strip-waveguide as bus waveguide. (**c**) Hybrid-waveguide ring resonator consisting of a slot- and strip waveguide. The strip-to-slot optical mode transition is achieved by a slow-varying waveguide taper. (© 2018 IEEE. Reprinted, with permission, from Ref. [14]).

As mentioned before, each waveguide-type has advantages and disadvantages and therefore, a design trade-off regarding sensitivity and optical losses is necessary. Recently, a hybrid-waveguide ring resonator was proposed to combine a strip waveguide with a slot waveguide in such a way that the figure of merit $FOM=S_{rr}/FWHM$ is maximized [14]. Figure 4 shows a schematic representation of this SOI ring resonator, which consists of both a strip waveguide and a slot waveguide [47,48,49,50]. This type of ring resonator has been demonstrated to have an improved figure of merit compared to more common strip or slot waveguide-based ring resonators, as it is summarized in Table 1.

A comparative study on the sensor performance of slot and strip waveguide ring resonators is given in Ref. [51]. Here, glucose level monitoring in blood samples in the range 10 to 200mg/dL using minimal invasive technique is simulated. Additionally, a six times higher $S_{rr}$ of the slot waveguide ring resonator is estimated using the Finite Element Method (FEM).

In 2009, a novel sensing approach were introduced by Daoxin Dai [52]. He proposed to cascade two micro-rings in such a way that it works analogously to a Vernier-scale. Claes et al. [53] have demonstrated this principle by using micro-rings with large circumferences to make it work in another regime that allows to reduce the detection limit. This method were several times adopted and highly sensitive biosensors were demonstrated that exceed the sensitivity of more common single-ring sensors [54,55,56].

It is also notable that advances on planar silicon ring resonators with novel guiding structures have been theoretically investigated. Such resonator structures show ring resonator sensitivities of up to 120nm/RIU and high Q-factors of $10^{5}$ [58], which could result in a record high FOM of about 7742. More recently, polarization independent slot waveguide structures were theoretically demonstrated to double the waveguide sensitivity [59]. More recently, in 2019, sub-wavelength grating (SWG) waveguides have been demonstrated to exhibit a sensitivity of 1900pm/nm [60]. These results show the potential for integrated high sensitive optical biosensors in a SOI technology and give prospective to further improvements.

## 3. Functionalization Procedures and Applications

In this section we give a basic introduction of to label-free functionalization procedures and a short overview of recent advances in the bio-functionalization of photonic sensors based on SOI-technology.

The aim of current research on SOI ring resonators is to improve their sensitivity in order to make them cost effective through the integration in highly scalable production flows and to realize real-time indication of biomolecules and toxins with high reliability and is currently focused primarily to medical diagnostics. Rapid and simplge diagnostics of acute inflammation, for example, can support the decision for the correct medicine to provide primary medical care inside and outside of hospitals as well as to monitor therapeutic interventions.

For experimental development antibody-antigen model systems like anti bovine serum albumin (antiBSA)-bovine serum albumin (BSA) [61] are typically used for proof of concepts. In a standard procedure (e.g., ELISA), the high specificity and affinity biotin-streptavidin biotin binding is widely used as linker. Therefore, this system is also used as model system for proof of concepts and to validate SOI ring resonators [45,62,63]. In general, the biospecific interaction is following the key-lock principle allowing for high selectivity.

Over the past 10 years, several researchers have successfully demonstrated functionalized SOI ring resonators for the detection of acute inflammations, viral diseases and cancer by biomarkers such as proteins [12,45,62], interleukins [64], nucleic acids [65,66], and viruses [67]. A brief overview is given in Table 2.

For this purpose, the silicon surface of the ring resonator has to be functionalized with the corresponding bioactive receptors. The bioactive receptors can be either covalently or adsorptively immobilized to the silicon surface.

**Figure 5:**
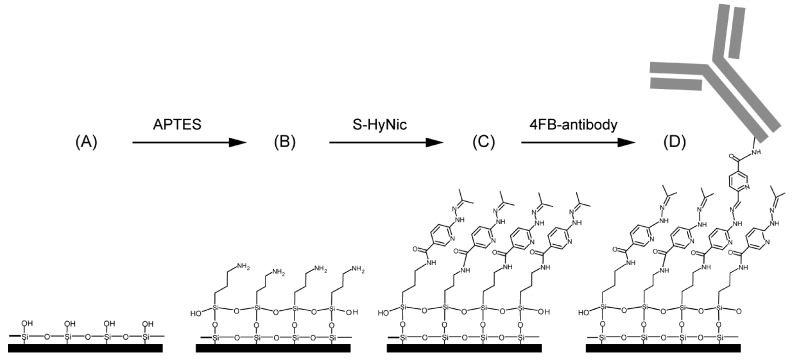
Representative example for surface functionalization. (**A**) Silicon surface of an activated SOI ring resonator. (**B**) In order to generate an amino-terminated surface, APTES is reacting with the surface siloxane groups. (**C**) Afterwards, S-HyNic is reacting with primary amines to build a HyNic-displaying surface. (**D**) Finally, the addition of 4FB-modified antibodies leads to a hydrazone bond formation between the 4FB moieties on the antibodies and the HyNic moieties on the surface. Reprinted (adapted) with permission from Ref. [72]. Copyright (2018) American Chemical Society.

Covalent immobilization gives a tight binding of the organic receptors on the inorganic silicon surface. As a rule, up to four reaction steps (A–D) are required for this, as shown in Figure 5 by means of an example from Ref. [72]:

**(A) Surface activation**
  The surface activation is carried out by cleaning the silicon surface with piranha solution or hydrogen peroxide-ammonium hydroxide solution followed by an argon plasma to generate hydroxyl groups.
**(B) Surface functionalization**
  To immobilize the bioactive receptors, agents like bifunctional organosilane of the general formula R${}_{3}$-Si-(CH${}_{2}$)${}_{n}$-X with hydrolysable groups R (OCH${}_{3}$, CH${}_{2}$CH${}_{3}$, Cl, F, SH) are often used, for example, (3-Aminopropyl)triethoxysilane (APTES) (in Figure 5). The choice of functional groups X (NH${}_{2}$, epoxy, SH, C=C) depends strongly on the desired specification. The condensation of these materials with the surface hydroxyl groups results in the formation of siloxane bonds (Si-O-Si). Such coupling leads to monolayers that is covalently bonded on the silicon surface and therefore highly stable.
**(C) Linker**
  The linker molecules are also bifunctional. In some cases they are symmetrical in structure, such as the widely used amine-to-amine linker glutaraldehyde or bis (sulfosuccinimidyl) suberate (BS3), but may also carry two different functional groups, e.g., *N*-γ-maleimidobutyryl oxysuccinimide ester (GMBS), which is an amine-to-sulfhydryl crosslinker that contains NHS esters and maleimide reactive groups at opposite ends of a short spacer arm. In the example shown in Figure 5, a heterobifunctional crosslinker reacts with the amino-modified surfaces. In this case, a succinimidyl-6-hydrazino-nicotinamide (S-HyNic) is used as linker molecule.
**(D) Immobilisation of receptor**
  Immobilization of biomolecules as receptors such as antibodies requires a pre-modification of those biomolecules. Thus, biotinylation can introduce functionality into the biomolecule. In the example shown in Figure 5, a 4-formylbenzamide (4FB)-modified antibody is used to form a stable covalent hydrazone linkage at the 6-hydrazinonicotinamide (HyNic) moieties.

On the one hand, this procedure allows a stable binding but, on the other hand, it requires a sophisticated synthetization, but the application justifies the effort.

**Figure 6:**
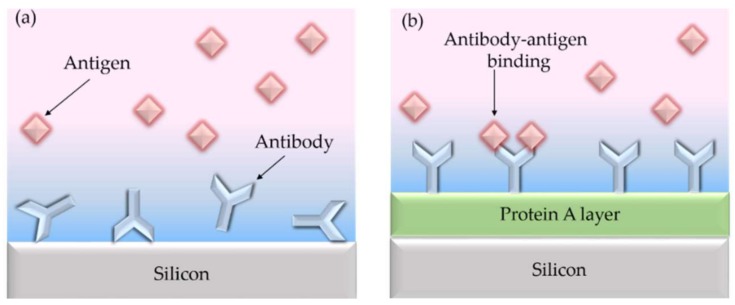
(**a**) The antibody receptors are usually randomly oriented on the silicon surface when they are directly immobilized using physical adsorption. (**b**) Using a protein A layer leads to properly oriented antibody receptors. Reproduced from Ref. [61] (CC BY 4.0).

In contrast, adsorptive immobilization via ionic or van der Waals interactions is easier to use, and allows for fast measurements without specific reagents and is applied despite the disadvantage of low sensitivity due to an incorrect orientation, as shown in Figure 6a. Using a protein layer, Caroselli and co-workers improved the alignment of antibody receptors [61].

However, adsorption is the weakest compound because it can be resolved by varying pH, temperature or ionic strength changes. Another problem is the possible inactivation due to the change in the 3D-structure of the biomolecule after adsorption on the sensor surface. For this reason, the covalent immobilization is preferred for highly sensitive and selective measurements.

SOI ring resonators are well suited for the detection of analytes with molecular weights in the range of kilodaltons, while molecular weights of more than megadalton (MDa) may exceed their size the evanescent field region of the sensor and lead to an invalid result [43]. However, recognition of bean pod mottle virus (7MDa) demonstrates the principle feasibility of detecting high molecular weight molecules. For small molecules [MDa] a detectable signal is difficult to obtain from SOI-based sensors, especially at low concentrations due to low sensitivity or high noise level.

One great advantage of integrated photonic biosensors is the ability for multiplexing making this technology attractive for diagnostics and interaction screening [73]. For example, Luchansky et al. have demonstrated a fast multiplexing system using 32-element arrays of ring resonators to quantify several species with excellent time-to-result [69,74]. In particular, they have detected the cytokines interleukin-2 (IL-2), interleukin-4 (IL-4), interleukin-5 (IL-5), and tumor necrosis factor alpha (TNFα) in parallel with high accuracy in serum-containing cell media.

Recent developments in antifouling coatings have led to a further reduction of nonspecific protein binding to the sensor surface. For example, Jäger et al. [75] have examined methylated dendritic polyglycerol (dPG(OMe)) as a protective layer. In this case, fibrinogen was used to test the antifouling properties. A reduction of 87% in the binding of fibrinogen to the silicon surface was demonstrated by using a SOI rib waveguide-based ring resonator.

## 4. System Integration

In this section we discuss different measurement set-ups used in laboratories and review current advances to integrate them into a SOI platform.

**Figure 7:**
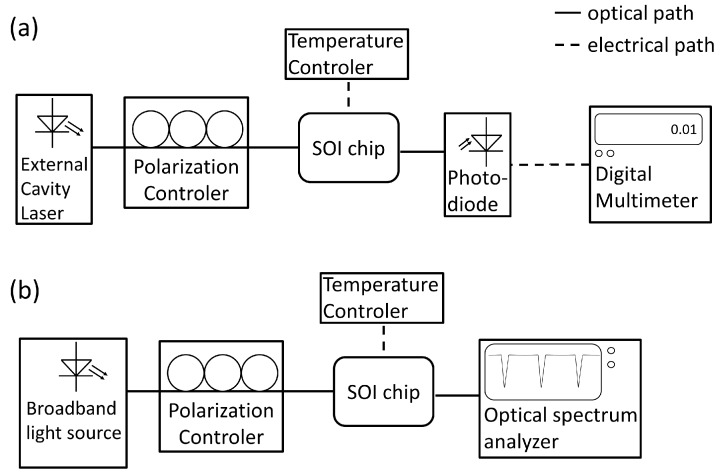
Schematic of typical measurement set-ups in laboratories. (**a**) The light source consists of an external cavity laser, which can tune its wavelength (tunable laser). In this case, a photodiode can be used as detector. (**b**) If a broad band light source (e.g., superluminescent diode) is employed, an optical spectrum analyzer is needed on the detector side.

The most common set-up is shown in Figure 7. It comprises either a tunable laser source in combination with a photodiode (Figure 7a) or a broadband light source with a optical spectrum analyzer (Figure 7b). The polarization of the light is typically adjusted by a 3-paddle polarization controller. To avoid temperature fluctuations, the sample holder is heated just above room temperature. The main disadvantage of this measurement is the light coupling since it requires a precise adjustment of optical fibers.

To avoid fiber coupling, current research in SOI technology is focusing on the integration of light sources and photodiodes. While Ge-photodiodes have been successfully integrated in a SOI platform [33,76,77,78], the integration of laser sources is still challenging [79]. Current approaches employ wafer-to-wafer [35,80] or die-to-wafer [31,81,82] bonding.

**Figure 8:**
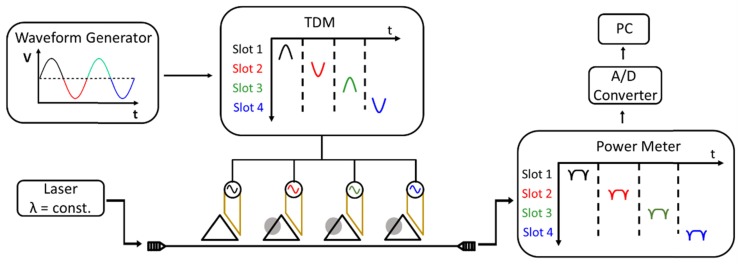
Schematic of ring resonator array. Each ring is separately addressed in the electronic regime to individually measure the transmission. The sinusoidal input signal is divided in certain time slots. Adopted from Ref. [85].

A novel integration scheme was recently proposed [83,84]. Here, a single wavelength laser is used in combination with a monolithically integrated Ge-photodiode. To obtain the transmission peaks, the ring resonator is tuned by employing a thermal heater. Both thermal tuning of the effective refractive index and thermo-optical multiplexing is used, while an expensive tunable laser source is avoided [85]. Figure 8 shows a schematic of this set-up. Each ring resonator is individually addressed and tunable by electronic signals. The modulation signal for the ring array is provided by a sinusoidal tuning signal and a separate switching unit that divides the signal in certain time slots, which are connected to a specific ring. The modulation signal induces a thermal refractive index change and, therefore, changes the resonance condition of the ring. In this way, the transmission of each ring can be detected without the need for a tunable laser.

Current integration issues are related to chip packaging. Since the integration of light sources, photodetectors and readout require conductive interconnects and occupy many space, a backend of line (BEOL) until five metal levels are necessary for a monolithic integration. This, on the other hand, requires a relatively deep etching procedure through the BEOL in order to release the sensing area (ring resonator). This leads to a high aspect ratio and makes surface functionalization and the implementation of micro-fluids challenging. Therefore, current integration approaches prefer a hybrid integration; i.e., the integration light source and detector unit on a separate chip. The disadvantage of this approach is the sophisticated optical interconnection between each chip such as, for example, photonic wire bonding realized by direct-write two-photon lithography [86,87,88]. Therefore, the system integration still requires further developments and is in the focus of current research to achieve high throughput.

## 5. Outlook

During the last two decades, integrated photonic sensors have been intensively studied in terms of sensitivity and reliability. However, the bottle-neck for a transfer from laboratory to industry is the position of the sensing area, since it adjoins optical and electronical components. This prohibits a full packaging and makes the sensor handling impractical. Towards the large-scale commercialization of SOI-based ring resonator biosensors, a low-cost and scalable packaging including a micro-fluidic system is required [89]. Recent developments on CMOS-compatible epoxy chip-in-carrier processes [90] or the fan-out wafer-level packaging process [4,91,92,93] have been demonstrated as good candidates towards this goal. Future work, however, should focus on further improvements of wafer-level packaging but also on monolithic integration of SOI ring resonators with a laser-source, photodetector and electrical interconnects on the same chip to provide full lab-on-a-chip solution.

## 6. Conclusions

Biosensors based on SOI ring resonators are reviewed and discussed. The theoretical background in terms of waveguide and resonator sensitivity as well as detection limits is provided and current developments in ring resonator geometries are reviewed. Furthermore, an overview of functionalized SOI ring resonators and their applications is provided. Finally, experimental set-ups for the optical characterization are described and current integration approaches are reviewed.

## Figures and Tables

**Table 1:** Comparison of different ring resonators based on SOI-technology. (© 2018 IEEE. Reprinted, with permission, from Ref. [14]).

	Slot-Waveguide	Strip-Waveguide	Hybrid-Waveguide
footprint [μm${}^{2}$]	130	100	2720
$S_{rr}$ [nm/RIU]	298	70	106.29
*Q*	330	20,000	18,500
FOM	63	903	1337
Ref.	[57]	[45]	[14]

**Table 2:** Examples of application and selection of biomolecules that have been detected by integrated photonic biosensors based on SOI ring resonators.

Application	Analyte/Biomarker	Receptor/Target	Detection Limit	Ref.
Acute inflammation	C-reactive protein (CRP)	Anti-CRP	6.5 pM	[64,68]
Acute inflammation	Interleukin 2,4,5	Anti-CRP	6–100 pM	[68,69]
HIV	Human immunoglobulin (Hu-IgG)	Anti-Hu-lgG	1 ng	[70]
Hepatitis	Human serum albumin	Anti-Albumin	3.4 pg	[45]
Meningitis	tmRNA	DNA	0.524nM	[66]
Prostate cancer	Prostate specific antigen (PSA)	Anti-PSA	0.4 nM	[12,71]
Liver cancer	α-fetoprotein (AFP)	Anti-AFP	100 pM	[12]
Bowel cancer	Carcinoembrionic antigen (CEA)	Anti-CEA	10 pM	[72]
Bladder cancer	Tumor necrosis factor (TNF)	Antibody	100 pM	[69]
Model system	Green fluorescent protein (GFP)	Antibody	0.1 mg/mL	[71]
Model system	Streptavidin	Biotin	60–150 fM	[45,62,63]
Food monitoring	Bean pod mottle virus	Antibody	1.43 pM	[67]

## References

[1] Thomson D., Zilkie A., Bowers J.E., Komljenovic T., Reed G.T., Vivien L., Marris-Morini D., Cassan E., Virot L., Fédéli J.M. (2016). Roadmap on silicon photonics. J. Opt..

[2] Chrostowski L., Hochberg M. (2015). Silicon Photonics Design: From Devices to Systems.

[3] Boeuf F., Cremer S., Temporiti E., Shaw M., Vulliet N., Ristoiu D., Farcy A., Pinguet T., Mekis A., Masini G. Recent progress in silicon photonics R&D and manufacturing on 300 mm wafer platform. Proceedings of the Optical Fiber Communication Conference.

[4] Laplatine L., Luan E., Cheung K., Ratner D.M., Dattner Y., Chrostowski L. (2018). System-level integration of active silicon photonic biosensors using Fan-Out Wafer-Level-Packaging for low cost and multiplexed point-of-care diagnostic testing. Sens. Actuators B Chem..

[5] Knoll D., Lischke S., Barth R., Zimmermann L., Heinemann B., Rucker H., Mai C., Kroh M., Peczek A., Awny A. High-performance photonic BiCMOS process for the fabrication of high-bandwidth electronic-photonic integrated circuits. Proceedings of the 2015 IEEE International Electron Devices Meeting (IEDM).

[6] Zhou Z., Yin B., Michel J. (2015). On-chip light sources for silicon photonics. Light Sci. Appl..

[7] Sakib M.N.N., Sun J., Kumar R., Driscoll J., Rong H. Demonstration of a 50 Gb/s all-silicon waveguide photodetector for photonic integration. Proceedings of the CLEO: QELS_Fundamental Science.

[8] Li S., Tarr G., Winnie N.Y. Monolithic integration of SOI waveguide photodetectors and transimpedance amplifiers. Proceedings of the Silicon Photonics XIII.

[9] Alimonti G., Ammendola R., Andreazza A., Badoni D., Bonaiuto V., Casalboni M., Matteis F.D., Mai A., Paoluzzi G., Prosposito P. (2018). Use of silicon photonics wavelength multiplexing techniques for fast parallel readout in high energy physics. Nucl. Instrum. Methods Phys. Res. Sect. A Accel. Spectrom. Detect. Assoc. Equip..

[10] Zang K., Zhang D., Huo Y., Chen X., Lu C.Y., Fei E.T., Kamins T.I., Feng X., Huang Y., Harris J.S. (2015). Microring bio-chemical sensor with integrated low dark current Ge photodetector. Appl. Phys. Lett..

[11] Williams E.H., Davydov A.V., Motayed A., Sundaresan S.G., Bocchini P., Richter L.J., Stan G., Steffens K., Zangmeister R., Schreifels J.A. (2012). Immobilization of streptavidin on 4H-SiC for biosensor development. Appl. Surf. Sci..

[12] Washburn A.L., Luchansky M.S., Bowman A.L., Bailey R.C. (2010). Quantitative, Label-Free Detection of Five Protein Biomarkers Using Multiplexed Arrays of Silicon Photonic Microring Resonators. Anal. Chem..

[13] Densmore A., Xu D.X., Janz S., Waldron P., Mischki T., Lopinski G., Delâge A., Lapointe J., Cheben P., Lamontagne B. (2008). Spiral-path high-sensitivity silicon photonic wire molecular sensor with temperature-independent response. Opt. Lett..

[14] Steglich P., Villringer C., Pulwer S., Heinrich F., Bauer J., Dietzel B., Mai C., Mai A., Casalboni M., Schrader S. (2017). Hybrid-Waveguide Ring Resonator for Biochemical Sensing. IEEE Sens. J..

[15] Guan X., Wang X., Frandsen L.H. (2016). Optical temperature sensor with enhanced sensitivity by employing hybrid waveguides in a silicon Mach-Zehnder interferometer. Opt. Express.

[16] Zhang Y., Zou J., He J.J. (2018). Temperature sensor with enhanced sensitivity based on silicon Mach-Zehnder interferometer with waveguide group index engineering. Opt. Express.

[17] Sakamoto H., Minpou Y., Sawai T., Enami Y., Suye S.I. (2016). A novel optical biosensing system using Mach–Zehnder-type optical waveguide for influenza virus detection. Appl. Biochem. Biotechnol..

[18] Rodriguez G.A., Hu S., Weiss S.M. (2015). Porous silicon ring resonator for compact, high sensitivity biosensing applications. Opt. Express.

[19] Xu W., Xie L., Zhu J., Xu X., Ye Z., Wang C., Ma Y., Ying Y. (2016). Gold Nanoparticle-Based Terahertz Metamaterial Sensors: Mechanisms and Applications. ACS Photonics.

[20] Ahmadivand A., Gerislioglu B., Tomitaka A., Manickam P., Kaushik A., Bhansali S., Nair M., Pala N. (2018). Extreme sensitive metasensor for targeted biomarkers identification using colloidal nanoparticles-integrated plasmonic unit cells. Biomed. Opt. Express.

[21] Ahmadivand A., Gerislioglu B., Manickam P., Kaushik A., Bhansali S., Nair M., Pala N. (2017). Rapid Detection of Infectious Envelope Proteins by Magnetoplasmonic Toroidal Metasensors. ACS Sens..

[22] Wang H., Zhou X., Liu Y., Yang H., Guo Q. (2010). Determination of aflatoxin M1 in milk by triple quadrupole liquid chromatography-tandem mass spectrometry. Food Addit. Contam. Part A.

[23] Paniel N., Radoi A., Marty J.L. (2010). Development of an electrochemical biosensor for the detection of aflatoxin M1 in milk. Sensors.

[24] Tothill I. (2011). Biosensors and nanomaterials and their application for mycotoxin determination. World Mycotoxin J..

[25] Markaki P., Melissari E. (1997). Occurrence of aflatoxin M1 in commercial pasteurized milk determined with ELISA and HPLC. Food Addit. Contam..

[26] Behfar A., Khorasgani Z.N., Alemzadeh Z., Goudarzi M., Ebrahimi R., Tarhani N. (2012). Determination of Aflatoxin M1 levels in produced pasteurized milk in Ahvaz City by using HPLC. Jundishapur J. Nat. Pharm. Prod..

[27] Parker C.O., Lanyon Y.H., Manning M., Arrigan D.W., Tothill I.E. (2009). Electrochemical immunochip sensor for aflatoxin M1 detection. Anal. Chem..

[28] Sibanda L., Saeger S.D., Peteghem C.V. (1999). Development of a portable field immunoassay for the detection of aflatoxin M1 in milk. Int. J. Food Microbiol..

[29] Ince R., Narayanaswamy R. (2006). Analysis of the performance of interferometry, surface plasmon resonance and luminescence as biosensors and chemosensors. Anal. Chim. Acta.

[30] Wang Y., Dostálek J., Knoll W. (2009). Long range surface plasmon-enhanced fluorescence spectroscopy for the detection of aflatoxin M1 in milk. Biosens. Bioelectron..

[31] Zhang J., Haq B., O’Callaghan J., Gocalinska A., Pelucchi E., Trindade A.J., Corbett B., Morthier G., Roelkens G. (2018). Transfer-printing-based integration of a III-V-on-silicon distributed feedback laser. Opt. Express.

[32] Komljenovic T., Davenport M., Hulme J., Liu A.Y., Santis C.T., Spott A., Srinivasan S., Stanton E.J., Zhang C., Bowers J.E. (2016). Heterogeneous silicon photonic integrated circuits. J. Lightw. Technol..

[33] Lischke S., Knoll D., Mai C., Zimmermann L., Peczek A., Kroh M., Trusch A., Krune E., Voigt K., Mai A. (2015). High bandwidth, high responsivity waveguide-coupled germanium p-i-n photodiode. Opt. Express.

[34] Shang J., Cheng F., Dubey M., Kaplan J.M., Rawal M., Jiang X., Newburg D.S., Sullivan P.A., Andrade R.B., Ratner D.M. (2012). An Organophosphonate Strategy for Functionalizing Silicon Photonic Biosensors. Langmuir.

[35] Duprez H., Descos A., Ferrotti T., Sciancalepore C., Jany C., Hassan K., Seassal C., Menezo S., Bakir B.B. (2015). 1310 nm hybrid InP/InGaAsP on silicon distributed feedback laser with high side-mode suppression ratio. Opt. Express.

[36] Megalini L., Bonef B., Cabinian B.C., Zhao H., Taylor A., Speck J.S., Bowers J.E., Klamkin J. (2017). 1550-nm InGaAsP multi-quantum-well structures selectively grown on v-groove-patterned SOI substrates. Appl. Phys. Lett..

[37] Taniguchi T., Hirowatari A., Ikeda T., Fukuyama M., Amemiya Y., Kuroda A., Yokoyama S. (2016). Detection of antibody-antigen reaction by silicon nitride slot-ring biosensors using protein G. Opt. Commun..

[38] Steglich P., You K.Y. (2018). Silicon-on-Insulator Slot Waveguides: Theory and Applications in Electro-Optics and Optical Sensing. Emerging Waveguide Technology.

[39] Steglich P., Villringer C., Dümecke S., Michel Y.P., Casalboni M., Schrader S. Silicon-on-insulator slot-waveguide design trade-offs. Proceedings of the 2015 International Conference on Photonics, Optics and Laser Technology (PHOTOPTICS).

[40] Steglich P., Villringer C., Pulwer S., Casalboni M., Schrader S., Ribeiro P., Raposo M. (2016). Design Optimization of Silicon-on-Insulator Slot-Waveguides for Electro-optical Modulators and Biosensors. Photoptics 2015.

[41] Steglich P., Villringer C., Dümecke S., Michel Y., Casalboni M., Schrader S. (2015). Design optimization of slot-waveguides covered with organic cladding materials for integrated photonic devices. NWK.

[42] Milvich J., Kohler D., Freude W., Koos C. (2018). Surface sensing with integrated optical waveguides: A design guideline. Opt. Express.

[43] Luan E., Shoman H., Ratner D.M., Cheung K.C., Chrostowski L. (2018). Silicon Photonic Biosensors Using Label-Free Detection. Sensors.

[44] Chrostowski L., Grist S., Flueckiger J., Shi W., Wang X., Ouellet E., Yun H., Webb M., Nie B., Liang Z. (2012). Silicon photonic resonator sensors and devices. Proc. SPIE.

[45] Vos K.D., Bartolozzi I., Schacht E., Bienstman P., Baets R. (2007). Silicon-on-Insulator microring resonator for sensitive and label-free biosensing. Opt. Express.

[46] White I., Zhu H., Suter J., Hanumegowda N.M., Oveys H., Zourob M., Fan X. (2007). Refractometric Sensors for Lab-on-a-Chip Based on Optical Ring Resonators. IEEE Sens. J..

[47] Steglich P., Mai C., Stolarek D., Lischke S., Kupijai S., Villringer C., Pulwer S., Heinrich F., Bauer J., Meister S. (2015). Novel Ring Resonator Combining Strong Field Confinement With High Optical Quality Factor. IEEE Photonics Technol. Lett..

[48] Steglich P., Mai C., Villringer C., Pulwer S., Casalboni M., Schrader S., Mai A. (2018). Quadratic electro-optic effect in silicon-organic hybrid slot-waveguides. Opt. Lett..

[49] Steglich P., Mai C., Stolarek D., Lischke S., Kupijai S., Villringer C., Pulwer S., Heinrich F., Bauer J., Meister S. (2016). Partially slotted silicon ring resonator covered with electro-optical polymer. Proc. SPIE.

[50] Steglich P., Mai C., Peczek A., Korndörfer F., Villringer C., Dietzel B., Mai A. Quadratic electro-optical silicon-organic hybrid RF modulator in a photonic integrated circuit technology. Proceedings of the 2018 IEEE International Electron Devices Meeting (IEDM).

[51] Singh R.R., Kumari S., Gautam A., Priye V. (2019). Glucose Sensing Using Slot Waveguide-Based SOI Ring Resonator. IEEE J. Sel. Top. Quantum Electron..

[52] Dai D. (2009). Highly sensitive digital optical sensor based on cascaded high-Q ring-resonators. Opt. Express.

[53] Claes T., Bogaerts W., Bienstman P. (2010). Experimental characterization of a silicon photonic biosensor consisting of two cascaded ring resonators based on the Vernier-effect and introduction of a curve fitting method for an improved detection limit. Opt. Express.

[54] Hoste J.W., Soetaert P., Bienstman P. (2016). Improving the detection limit of conformational analysis by utilizing a dual polarization Vernier cascade. Opt. Express.

[55] Jiang X., Ye J., Zou J., Li M., He J.J. (2013). Cascaded silicon-on-insulator double-ring sensors operating in high-sensitivity transverse-magnetic mode. Opt. Lett..

[56] Liu Y., Li Y., Li M., He J.J. (2017). High-sensitivity and wide-range optical sensor based on three cascaded ring resonators. Opt. Express.

[57] Claes T., Molera J., De Vos K., Schachtb E., Baets R., Bienstman P. (2009). Label-Free Biosensing with a Slot-Waveguide-Based Ring Resonator in Silicon on Insulator. IEEE Photonics J..

[58] Ciminelli C., Dell’Olio F., Conteduca D., Campanella C., Armenise M. (2014). High performance SOI microring resonator for biochemical sensing. Opt. Laser Technol..

[59] Pan C., Rahman B.M.A. (2017). High-Sensitivity Polarization-Independent Biochemical Sensor Based on Silicon-on-Insulator Cross-Slot Waveguide. IEEE J. Sel. Top. Quantum Electron..

[60] Luan E., Yun H., Laplatine L., Dattner Y., Ratner D.M., Cheung K.C., Chrostowski L. (2019). Enhanced Sensitivity of Subwavelength Multibox Waveguide Microring Resonator Label-Free Biosensors. IEEE J. Sel. Top. Quantum Electron..

[61] Caroselli R., García Castelló J., Escorihuela J., Bañuls M.J., Maquieira A., García-Rupérez J. (2018). Experimental Study of the Oriented Immobilization of Antibodies on Photonic Sensing Structures by Using Protein A as an Intermediate Layer. Sensors.

[62] Iqbal M., Gleeson M.A., Spaugh B., Tybor F., Gunn W.G., Hochberg M., Baehr-Jones T., Bailey R.C., Gunn L.C. (2010). Label-Free Biosensor Arrays Based on Silicon Ring Resonators and High-Speed Optical Scanning Instrumentation. IEEE J. Sel. Top. Quantum Electron..

[63] Xu D.X., Densmore A., Delâge A., Waldron P., McKinnon R., Janz S., Lapointe J., Lopinski G., Mischki T., Post E. (2008). Folded cavity SOI microring sensors for high sensitivity and real time measurement of biomolecular binding. Opt. Express.

[64] Luchansky M.S., Washburn A.L., McClellan M.S., Bailey R.C. (2011). Sensitive on-chip detection of a protein biomarker in human serum and plasma over an extended dynamic range using silicon photonic microring resonators and sub-micron beads. Lab Chip.

[65] Qavi A.J., Kindt J.T., Gleeson M.A., Bailey R.C. (2011). Anti-DNA:RNA Antibodies and Silicon Photonic Microring Resonators: Increased Sensitivity for Multiplexed microRNA Detection. Anal. Chem..

[66] Scheler O., Kindt J.T., Qavi A.J., Kaplinski L., Glynn B., Barry T., Kurg A., Bailey R.C. (2012). Label-free, multiplexed detection of bacterial tmRNA using silicon photonic microring resonators. Biosens. Bioelectron..

[67] McClellan M.S., Domier L.L., Bailey R.C. (2012). Label-free virus detection using silicon photonic microring resonators. Biosens. Bioelectron..

[68] Luchansky M.S., Bailey R.C. (2010). Silicon photonic microring resonators for quantitative cytokine detection and T-cell secretion analysis. Anal. Chem..

[69] Luchansky M.S., Bailey R.C. (2011). Rapid, Multiparameter Profiling of Cellular Secretion Using Silicon Photonic Microring Resonator Arrays. J. Am. Chem. Soc..

[70] Vos K.D., Girones J., Claes T., Koninck Y.D., Popelka S., Schacht E., Baets R., Bienstman P. (2009). Multiplexed Antibody Detection With an Array of Silicon-on-Insulator Microring Resonators. IEEE Photonics J..

[71] Fukuyama M., Nishida M., Abe Y., Amemiya Y., Ikeda T., Kuroda A., Yokoyama S. (2011). Detection of Antigen–Antibody Reaction Using Si Ring Optical Resonators Functionalized with an Immobilized Antibody-Binding Protein. Jpn. J. Appl. Phys..

[72] Washburn A.L., Gunn L.C., Bailey R.C. (2009). Label-Free Quantitation of a Cancer Biomarker in Complex Media Using Silicon Photonic Microring Resonators. Anal. Chem..

[73] Kindt J.T., Bailey R.C. (2013). Biomolecular analysis with microring resonators: applications in multiplexed diagnostics and interaction screening. Curr. Opin. Chem. Biol..

[74] Shia W.W., Bailey R.C. (2012). Single domain antibodies for the detection of ricin using silicon photonic microring resonator arrays. Anal. Chem..

[75] Jäger M., Becherer T., Bruns J., Haag R., Petermann K. (2016). Antifouling coatings on SOI microring resonators for bio sensing applications. Sens. Actuators B Chem..

[76] Li G., Luo Y., Zheng X., Masini G., Mekis A., Sahni S., Thacker H., Yao J., Shubin I., Raj K., Cunningham J.E., Krishnamoorthy A.V. (2012). Improving CMOS-compatible Germanium photodetectors. Opt. Express.

[77] Fard M.M.P., Cowan G., Liboiron-Ladouceur O. (2016). Responsivity optimization of a high-speed germanium-on- silicon photodetector. Opt. Express.

[78] Chen H., Verheyen P., Heyn P.D., Lepage G., Coster J.D., Balakrishnan S., Absil P., Yao W., Shen L., Roelkens G. (2016). −1 V bias 67 GHz bandwidth Si-contacted germanium waveguide p-i-n photodetector for optical links at 56 Gbps and beyond. Opt. Express.

[79] Bo R., Yan H., Yanan L. (2016). Research progress of III–V laser bonding to Si. J. Semicond..

[80] Szelag B., Hassan K., Adelmini L., Ghegin E., Rodriguez P., Bensalem S., Nemouchi F., Bria T., Brihoum M., Brianceau P. Hybrid III-V/Si DFB laser integration on a 220 mm fully CMOS-compatible silionn photonlcsplotform. Proceedings of the 2017 IEEE International Electron Devices Meeting (IEDM).

[81] Juvert J., Cassese T., Uvin S., de Groote A., Snyder B., Bogaerts L., Jamieson G., Campenhout J.V., Roelkens G., Thourhout D.V. (2018). Integration of etched facet, electrically pumped, C-band Fabry-Pérot lasers on a silicon photonic integrated circuit by transfer printing. Opt. Express.

[82] Uvin S., Kumari S., Groote A.D., Verstuyft S., Lepage G., Verheyen P., Campenhout J.V., Morthier G., Thourhout D.V., Roelkens G. (2018). 1.3 *μ*m InAs/GaAs quantum dot DFB laser integrated on a Si waveguide circuit by means of adhesive die-to-wafer bonding. Opt. Express.

[83] Jäger M., Bruns J., Ehrentreich-Förster E., Petermann K. (2013). Arrays of Individually Addressable SOI Micro Ring Resonators for Bio Sensing. Advanced Photonics 2013.

[84] Jäger M., Volkmann D., Bruns J., Petermann K. (2015). Multiplexed Single Wavelength Bio Sensor for Low Cost Applications. Advanced Photonics 2015.

[85] Moock P., Kasper L., Jäger M., Stolarek D., Richter H., Bruns J., Petermann K. (2018). TDM-controlled ring resonator arrays for fast, fixed-wavelength optical biosensing. Opt. Express.

[86] Billah M.R., Blaicher M., Hoose T., Dietrich P.I., Marin-Palomo P., Lindenmann N., Nesic A., Hofmann A., Troppenz U., Moehrle M. (2018). Hybrid integration of silicon photonics circuits and InP lasers by photonic wire bonding. Optica.

[87] Lindenmann N., Balthasar G., Hillerkuss D., Schmogrow R., Jordan M., Leuthold J., Freude W., Koos C. (2012). Photonic wire bonding: a novel concept for chip-scale interconnects. Opt. Express.

[88] Gu Z., Amemiya T., Ishikawa A., Hiratani T., Suzuki J., Nishiyama N., Tanaka T., Arai S. (2015). Optical transmission between III–V chips on Si using photonic wire bonding. Opt. Express.

[89] Li H., Liu X., Li L., Mu X., Genov R., Mason A. (2017). CMOS electrochemical instrumentation for biosensor microsystems: A review. Sensors.

[90] Li L., Yin H., Mason A.J. (2018). Epoxy Chip-in-Carrier Integration and Screen-Printed Metalization for Multichannel Microfluidic Lab-on-CMOS Microsystems. IEEE Trans. Biomed. Circuits Syst..

[91] Laplatine L., Al’Mrayat O., Luan E., Fang C., Rezaiezadeh S., Ratner D., Cheung K., Dattner Y., Chrostowski L. (2017). System-level integration of active silicon photonic biosensors. Microfluidics, BioMEMS, and Medical Microsystems XV, Proceedings of the SPIE BiOS, San Francisco, CA, USA, 28 January–2 February 2017.

[92] Kaynak M., Wietstruck M., Göritz A., Wipf S.T., Inac M., Cetindogan B., Wipf C., Kaynak C.B., Wöhrmann M., Voges S. 0.13-μm SiGe BiCMOS technology with More-than-Moore modules. In Proceedings of the 2017 IEEE Bipolar/BiCMOS Circuits and Technology Meeting (BCTM).

[93] Braun T., Raatz S., Maass U., van Dijk M., Walter H., Hölck O., Becker K., Töpper M., Aschenbrenner R., Wöhrmann M. Development of a Multi-project Fan-Out Wafer Level Packaging Platform. Proceedings of the 2017 IEEE 67th Electronic Components and Technology Conference (ECTC).

